# Psychotic-Like Experiences: A Challenge in Definition and Assessment

**DOI:** 10.3389/fpsyt.2021.582392

**Published:** 2021-03-29

**Authors:** Barbara Hinterbuchinger, Nilufar Mossaheb

**Affiliations:** Clinical Division of Social Psychiatry, Department of Psychiatry and Psychotherapy, Medical University of Vienna, Vienna, Austria

**Keywords:** psychotic-like experiences, psychosis continuum, subclinical psychosis, psychosis phenotype, psychotic disorder

## Abstract

Assuming a continuum between psychotic experiences and psychotic symptoms aligned between healthy individuals and patients with non-psychotic and psychotic disorders, recent research has focused on subclinical psychotic experiences. The wide variety of definitions, assessment tools, and concepts of psychotic-like experiences (PLEs) might contribute to the mixed findings concerning prevalence and persistence rates and clinical impact. In this narrative review, we address the panoply of terminology, definitions, and assessment tools of PLEs and associated concerns with this multitude. Moreover, the ambiguous results of previous studies regarding the clinical relevance of PLEs are described. In conclusion, we address clinical implications and highly suggest conceptual clarity and consensus concerning the terminology and definition of PLEs. The development of an agreed upon use of a “gold standard” assessment tool seems essential for more comparable findings in future research.

## Introduction

In the last decades, contrary to the categorical approach of the “Kraepelinian dichotomy” (1), research has hypothesized a dimensional approach toward psychosis assuming a continuum of psychotic experiences and symptoms aligned between clinical and non-clinical populations (2). The assumption of a temporal and phenomenological psychosis continuum resulted in the examination of psychotic-like experiences (PLEs) in non-help-seeking individuals from the general population (3) to psychotic symptoms in individuals with “non-psychotic” disorders as well as manifest psychotic disorders in individuals with schizophrenia-spectrum disorders (4). Moreover, the concept of “at-risk mental state” (ARMS), and as a result, the implementation of operationalized criteria for the detection of individuals at ultra-high risk of psychosis (UHR) are well-established in clinical and research scope nowadays (5–7). Schizotypy, defined as a combination of personality traits with symptoms and experiences similar but not identical in intensity and phenomenology to schizophrenia spectrum disorders, was suggested as another endophenotype on the spectrum toward psychosis (8, 9).

The concept of psychosis as a transdiagnostic and extended phenotype (10) has resulted in the discussion about the redefinition of the concept and name of schizophrenia (11) and, moreover, in the examination of subclinical psychotic experiences (12–14) and the identification of PLEs as early indicators of psychosis onset (15, 16). However, the variety of definitions and assessment tools of PLEs has been postulated to contribute to the discrepancy of findings regarding prevalence rates (17–20) and persistence rates (21–24). Thus, in this narrative review, we aim to address the challenge concerning the variety of definitions and assessment tools regarding PLEs.

## The Definition of Psychotic-Like Experiences

Regarding the definition and terminology of PLEs, comparable confusion was reported as described for different concepts of ARMS/UHR (19, 25, 26): Originally, the term PLE was used for “subschizophrenic” symptoms located on a continuum ranging from normal experiences to “genuine” psychotic symptoms including hallucinations and delusions (27). A recently developed and widely used definition of PLEs describes them as “psychotic symptoms in the absence of illness” (28). Others referred to PLEs as psychotic symptoms in non-clinical populations (3), a “subclinical psychosis phenotype” (29) or referred to doubts about their true psychotic nature due to an uncertainty about the validity of their assessment (30). Van Os et al. differentiated between subclinical psychotic experiences and subclinical psychotic symptoms, whereas the latter were associated with distress and help-seeking behavior but not necessarily with a clinical psychotic disorder (17). In a recent systematic review, three main approaches regarding the definition of PLEs were described: PLEs defined by preset criteria, PLEs defined by assessment tools with a predetermined threshold, and PLEs defined by assessment tools without a predetermined threshold or criteria (19). The authors stated that the majority of papers defined PLEs quantitatively using widely varying assessment tools without specific phenomenological definitions, which might contribute to mixed findings in research (19). Preti et al. (31) differentiated between broadly and narrowly defined PLEs: While the former are defined as incidental and non-distressful unusual subjective experiences with uncertain appraisal, narrowly defined PLEs were suggested to be distressful unusual subjective experiences appraised with certainty. The authors stated that the expression “psychotic-like experiences” might be overinclusive and misleading and suggested the use of the expression “unusual subjective experiences” (USEs) aiming to avoid stigmatization.

Associated phenomena are anomalous self-experiences (ASEs) defined as disturbances in the subjective experiences of the self (32, 33) and described as first symptoms to appear in the prodrome predicting developing psychosis (34). Phenomenologically, these disturbances of self-affection result in a lack of own identity, distance between self and experience, and an alienation from the shared experience within a social context (35, 36). The similar concept of basic symptoms, often described as integral part of the anomalous self-experiences (37, 38), defines symptoms that are subtle, subjectively, and subclinically experienced disturbances in drive, affect, thinking, speech, perception, motor action, central vegetative functions, and stress tolerance also regarded as earliest symptoms within the development of psychosis (39, 40).

Overall, until now, there is no general consensus concerning the definition and conceptualizations of PLEs or associated phenomena, which may result or at least contribute to inconsistent findings and problems concerning the interpretation of results.

## The Assessment of Psychotic-Like Experiences

In this section, we aim to address various concerns associated with different aspects of PLE assessment tools. First of all, one major concern relates to the assessment of PLEs in the general population with self-report questionnaires, since a majority of individuals who rate positive for PLEs with self-scoring instruments could be identified as false positive after clinical assessment (41). Schultze-Lutter et al. reported a several-fold overestimation of the prevalence of clinician-rated psychotic symptoms when PLEs were assessed with self-report instruments including Peters et al. Delusion Inventory (PDI) and the revised Launay–Slade Hallucination Scale (LSHS-R) (42). Mixed results concerning the validity of PLE screening instruments were also found in adolescent populations (43, 44). However, self-reported subthreshold psychotic experiences in epidemiological non-help-seeking samples were found to index risk for the development of later psychotic disorders (45). Moreover, even “false positive” psychotic experiences were found to be associated with the later development of psychotic disorders (46), clinically relevant psychotic symptoms, mood and anxiety disorders, and reduced functioning (47). Second, there is a large heterogeneity concerning the inquired symptoms of PLE assessment tools as well as the measured “outcome” and inferences resulting from the answered items/questions: While the Magical Ideation Scale (MIS) examines magical ideation defined as an indicator of schizotypy and schizophrenia proneness (48), the Community Assessment of Psychic Experiences (CAPE), one of the most frequently used PLE self-rating instruments, was developed to assess the lifetime prevalence of PLEs in the general population by examining subclinical positive, negative, and depressive symptoms (49). Different questionnaires have different interpretations of PLEs leading to different findings regarding prevalence rates, persistence rates, and prognosis of PLEs. While most PLEs assessment tools assessed both thoughts *and* perceptions, some only assessed either thoughts *or* perceptions. Associated psychological factors including distress were assessed only in a few of the studied assessment tools (19). Needless to say, that related or phenomenologically similar phenomena as anomalous self-experiences (ASEs) are measured with separate assessment tools including the Inventory for Psychotic-Like Anomalous Experiences (IPASE) or the Examination of Anomalous Self-Experiences (EASE) (34, 50).

New approaches aiming for more subtle signs of reality distortion have created assessment tools for “exceptional experiences” (EEs) defined as deviations from experiences consistent with typical “reality models” (51) including hearing voices of beloved dead ones, déjà-vus, or out-of-body experiences (52). While EEs were first assumed as PLEs at the healthy end of the psychosis spectrum, the association with psychological problems (52) might need further clarification (13). Until now, there is no consensus about an agreed upon “gold standard” PLE assessment tool, and the multitude of assessment tools examining PLEs is striking. In Table 1, we give an overview of screening instruments for PLEs, psychotic symptoms, and high risk for psychosis.

**Table 1:** Screening instruments for psychotic-like experiences/psychosis/high risk for psychosis.

Community Assessments of Psychic Experiences (CAPE) (49, 53)	Subclinical positive, negative and depressive symptoms	Self-report assessment of psychotic experiences in the general population	Good reliability and validity; high sensitivity and specificity; helpful in screening clinical help-seeking individuals for UHR in a setting without specifically trained interviewers for the detection of UHR individuals	No disorganization dimension included
Composite Psychosis Risk Questionnaire–15-item version (54)	Interpersonal difficulty/social anxiety symptoms, depreciating descriptions, negative symptoms, and subthreshold psychotic-like experiences	15-item self-report prodromal screening questionnaire with less emphasis on attenuated psychotic symptoms and predictive values	Handy tool for increasing awareness and referral as first step risk assessment	Sensitivity and specificity of the final screening formula are 0.736 and 0.679, which are slightly lower than the original values; not developed as a better and more accurate solution for screening ultra-high risk subjects, but rather quick self-evaluation and referral, not specifically emphasizing the high likelihood of the transition to psychosis, but rather addressing a need for clinical attention
Early Detection Primary Care Checklist (PCCL) (55)	20-item checklist designed to identify young people at ultra-high risk of developing psychosis	Developed specifically for use by primary care practitioners to use within a help-seeking population	Excellent sensitivity (96%); quick and easy to use tool administered by primary care practitioners to help identify young people who may be in the early stages of psychosis and to make speedy and confident referrals to specialist services	Poor specificity (10%); not designed as diagnostic instrument or as population wide screens. A screen positive result indicates only the need for a further specialist assessment
Early Recognition Inventory Checklist/Inventory (ERIraos) (56–58)	Presence/absence of unspecific symptoms (checklist) and of late prodromal and psychotic symptoms during the last 12 months, and its intensity: 15-item screening Checklist and 50-item Symptom List	Available as questionnaire and interview; low-threshold screening instrument for people who have approached general practitioners or counseling services because of mental health problems, checklist assesses a contact to one of the early intervention centers should be made for detailed assessment; potential identification of at-risk persons at the earliest possible stage	Might contribute to higher accuracy of the referral process more detailed assessment; permits early recognition of psychosis risk in three steps of decreasing sensitivity and increasing specificity; translated into several foreign languages; relatively simple and practical to administer with high predictive power for psychosis onset	Checklist has broad symptom evaluation and is rather unspecific
PRIME Screen/PRIME Screen Revised (PS-R) (Prevention through Risk Identification, Management, and Education) (59)	12 questions assessing prodromal symptoms pf psychosis; PR assessment of “duration of symptoms” to the PRIME Screen;	Short self-administered questionnaire based on the positive symptom portion of the SIPS; useful screening tool for alerting clinicians to subjects with psychotic prodromal symptoms, advised for both general practice and clinical settings	Requires minutes to complete; fair to strong measures of validity; clinical construct validity shows that the screening test could sufficiently differentiate a clinical sample from a non-clinical population in the PS-R; excellent sensitivity (100%) and a good specificity (74%) in the PS-R	PRIME Screen was not validated in a non-clinical population to our knowledge; PS-R moderate concordant validity (43%) against the SIPS
PROD-Screen (60)	Prodromal symptoms including 29 questions assessing performance and symptoms	Instrument for screening prodromal symptoms indicating risk for psychotic conversion in the near future	Distinguishing prodromal from non-prodromal subjects with reasonable sensitivity (80%) and specificity (75%) in an epidemiologically mixed sample; useful tool for screening prodromal symptoms of psychosis and selecting subjects for more extensive research interviews	In clinical samples of psychiatric patients, PROD-screen cannot differentiate between SIPS-positive and SIPS-negative cases
Prodromal Questionnaire (PQ) (61)/Prodromal Questionnaire—Brief Version (62, 63)	Prodromal and psychotic symptoms	Screening in clinical high-risk and early psychosis research clinics and not outside of mental health settings for the following reasons; tool to preselect patients for more intensive interviewing	Good preliminary validity in detecting individuals with an interview-diagnosed prodromal or psychotic syndrome; in the PQ-B Version three or more positive differentiated between prodromal syndrome and psychotic syndrome diagnoses on the SIPS vs. those with no SIPS diagnoses with 89% sensitivity, 58% specificity; PQ-B is an effective, efficient self-report screen for prodromal psychosis when followed by diagnostic interview in a two-stage evaluation process in help-seeking population	PQ is time consuming for routine screening because of the long administration time; relatively low specificity; low sensitivity to the threshold between prodromal and manifest psychosis
Psychosis Screening Questionnaire (PSQ) (64)	Psychotic experiences	Psychotic experiences in non-clinical subjects, intended to screen for psychotic experiences	Brief measurement assessing only five psychotic symptoms	No assessment of the precise nature of the experiences
16-Item Version of the Prodromal Questionnaire (PQ-16) (65)	Nine items out of the perceptual abnormalities/hallucinations subscale, five items including unusual thought content/delusional ideas/paranoia, and two negative symptoms	Routine use in secondary mental health care and screening in large help-seeking populations	Good self-report screening instrument for use in secondary mental healthcare services to select subjects for interviewing for psychosis risk; appropriate for screening large help-seeking populations due to low number of items	Not sensitive enough to distinguish between UHR syndromes and psychosis
Self-Screen-Prodrome (SPro) and SPro-Psy-Risk (66, 67)	Ideas of being persecuted, concentration difficulties, increased sensitivity, depressed mood, and incipient changes in perception	32-item self-report screening instrument for general population groups to differentiate between healthy individuals, individuals with psychosis or at CHR, and patients with other psychiatric diagnoses	The total score of the SPro distinguishes between outpatients with a mental disorder and healthy individuals with a sensitivity of 85 % and a specificity of 91 %; six items of the SPro selected as a sub-scale (SPro-Psy-Risk) to distinguish individuals with psychosis or at clinical high risk (CHR) from outpatients with other ICD-10 diagnoses: with a sensitivity of 85 % and a specificity of 39 %	

## PLEs and Clinical Relevance

Albeit generally assumed as non-clinical phenomena, PLEs have been identified as early indicators of psychosis (15, 68, 69) forecasting the development of a psychotic disorder with a 4-fold increased risk in non-help-seeking individuals (45). Moreover, PLEs were identified as markers for risk of suicidal behavior and severe psychopathology including multi-morbidity and poor functioning (70). However, clinical relevance was shown to be associated with different subtypes of PLEs and the level of associated distress, need for treatment (71, 72), impact on comorbidities, functioning, and vulnerability toward psychosis (72–74). While the subdimension of persecutory ideation (72) and hallucinations, delusions, and paranoia (71) were strongly correlated with distress, subdimensions of grandiosity and paranormal beliefs did not correlate with distress and general measures of psychopathology (71).

Since negative associations were reported between some specific PLE subtypes and psychopathological dimensions as depression (75) and physical anhedonia (76), in line with the concept of the “healthy schizotype” (77), some specific PLE subtypes might not be associated with clinical impact but are being discussed to constitute a coping mechanisms and response helping to maintain mental functioning (78).

## Conclusion and Clinical Implications

In line with other researchers, we support the idea of conceptual clarity, consensus, and clear definitions regarding PLEs and associated concepts (9, 13, 25).

As previously suggested, an empirically established and agreed upon consensus catalog of terms, definitions, criteria, and categorization of PLEs according to psychopathological significance can help to achieve more standardized research and comparable data (25). To develop consensus, future research aiming for a better understanding of the phenomenology of PLEs seems crucial, especially since most studies did not focus on phenomenology of PLEs but rather quantitative measures (19). Since different subtypes of PLEs vary widely according to distress, associated psychopathology, help seeking, and clinical outcome (72, 79, 80), further research on the heterogenous character of different PLEs and associated psychological factors might help to differ between subclinical and clinically relevant psychotic experiences, improve risk screening, and foster new treatment and prevention strategies. The analysis of mediating factors between PLEs and clinical relevance including resilience, persistence of PLEs, environmental exposures, trauma, stressful life events, and cognitive impairments events might contribute to a better understanding of the evolution of mental disorders, especially psychosis spectrum disorders (23, 81–83). Moreover, the development and agreed upon use of a or a few “gold standard” assessment tools might help to gather more comparable data on PLEs concerning prevalence rates, prognosis, and other outcomes. As stated by other authors (13), longitudinal studies on PLEs and especially different PLE subtypes, might help to give more insight into the question why some individuals with PLEs develop mental disorders and others stay at the healthy end of the psychosis spectrum. Consequently, untangling this entanglement of PLE terms, definitions, and assessment tools might result in gathering more knowledge toward new prevention strategies and treatment approaches.

Well-validated screening instruments might help to detect distressed people with PLEs seeking help in general medical practice or in non-specialized psychiatric services. These may then be further assessed, in a clinical interview, in order to differentiate between actual psychotic symptoms or a clinical high-risk syndrome or PLEs without reaching any of the above. Finally, as part of a future consensus, not only definitions and assessments should be agreed upon but also the semantics used: Psychotic experiences in individuals from the healthy general population without associated distress should not be termed as symptoms (or “psychotic symptoms in the absence of illness,” which is in itself an oxymoron) (28) but as experiences. Whereas, in people with PLEs associated with distress, help-seeking behavior or any–not necessarily psychotic–psychiatric disorder, it is suggested that they be termed as symptoms. Some also argue that the term “psychotic” should not be used when referring to individuals fulfilling criteria for UHR/CHR, since only a minority of those continue to experience an actual psychotic disorder (84). We propose for the relevant proponents to engage in a continuing discussion aiming for an evidence-based consensus.

## Author Contributions

BH contributed to the concept of the review, the literature search, and draft of the manuscript. NM contributed to the concept of the review, supervision, and writing of the manuscript. All authors contributed to the article and approved the submitted version.

## Conflict of Interest

The authors declare that the research was conducted in the absence of any commercial or financial relationships that could be construed as a potential conflict of interest.
